# How to Predict the Weather

**Published:** 1874-04

**Authors:** 


How to Predict the Weather.—The following lucid directions for self-training as a weather prophet are not, we believe, from the Bureau over which "Old Probabilities" presides, but they bear internal evidence of coming from some high scientific authority: "If you wish to know whether it is going to storm or not, all you have to do is to find the storm vortex and see which side of it is the most moist. Multiply this by the square of the latent heat, subtract the time of day, and divide by the weathercock. The result will be the rarefication, plus the thermometric evolution of the north pole, and then a wayfaring man, though a natural know-nothing, can tell what will follow."

